# Chloroquine Affects Presynaptic Membrane Retrieval in Diaphragm Neuromuscular Junctions of Old Mice

**DOI:** 10.3390/ijms26010043

**Published:** 2024-12-24

**Authors:** Sepideh Jahanian, Chloe I. Gulbronson, Heather M. Gransee, Elena Millesi, Gary C. Sieck, Carlos B. Mantilla

**Affiliations:** 1 Department of Anesthesiology & Perioperative Medicine, Mayo Clinic, Rochester, MN 55905, USA; 2 Department of Surgery Research Services, Mayo Clinic, Rochester, MN 55905, USA; 3 Department of Physiology & Biomedical Engineering, Mayo Clinic, Rochester, MN 55905, USA

**Keywords:** autophagy, aging, synaptic vesicle recycling, neuromuscular dysfunction

## Abstract

Aging disrupts multiple homeostatic processes, including autophagy, a cellular process for the recycling and degradation of defective cytoplasmic structures. Acute treatment with the autophagy inhibitor chloroquine blunts the maximal forces generated by the diaphragm muscle, but the mechanisms underlying neuromuscular dysfunction in old age remain poorly understood. We hypothesized that chloroquine treatment increases the presynaptic retention of the styryl dye FM 4-64 following high-frequency nerve stimulation, consistent with the accumulation of unprocessed bulk endosomes. Diaphragm-phrenic nerve preparations from 24-month-old male and female C57BL/6 × 129 J mice were incubated with FM 4-64 (5 µM) and either chloroquine (50 µM) or vehicle during 80 Hz phrenic nerve stimulation. Acute chloroquine treatment significantly decreased FM 4-64 intensity at diaphragm neuromuscular junctions following 80 Hz phrenic nerve stimulation, consistent with disrupted synaptic vesicle recycling. A similar reduction was evident in regions with the greatest FM 4-64 fluorescence intensity, which most likely surround synaptic vesicle release sites. In the absence of nerve stimulation, chloroquine treatment significantly increased FM 4-64 intensity at diaphragm neuromuscular junctions. These findings highlight the importance of autophagy in regulating presynaptic vesicle retrieval (including vesicle recycling and endosomal processing) and support the role of autophagy impairments in age-related neuromuscular dysfunction.

## 1. Introduction

Aging is associated with time-related biochemical and functional changes, leading to decreased quality of life and eventual death [1]. Aging-related respiratory muscle dysfunction is an important predisposing factor for pneumonia in elderly patients [2]. Aging-related neuromuscular dysfunction is evident in respiratory muscles, including the diaphragm, and is associated with the loss of larger size phrenic motor neurons [3,4], diaphragm muscle sarcopenia [5,6,7], increased fragmentation [8,9], and decreased overlap of presynaptic and postsynaptic structures at diaphragm neuromuscular junctions, and a reduction in active synaptic areas [9]. Additionally, neuromuscular transmission failure of the diaphragm muscle [10,11,12], as well as a decline in the maximal forces generated during phrenic nerve stimulation [13,14], has been documented in old age, indicating a decreased functional capacity of the diaphragm muscle.

Autophagy is a highly regulated process for the degradation and sequestration of altered proteins and organelles within the cell [15]. The role of autophagy in aging-related neurodegeneration is becoming more evident [16,17]. Recycling of vesicle components and disposing of damaged structures by autophagy is necessary to maintain efficient synaptic transmission at the neuromuscular junction [18].

Chloroquine is an antimalarial drug that inhibits autophagosome–lysosome fusion during the degradation phase of autophagy and thus can be used in experiments to mimic autophagy inhibition [15,19]. In mouse phrenic nerve–hemidiaphragm preparations, chloroquine was also shown to reduce motor end plate potential and quantal content release by nerve stimulation (≥50 µM for 45 min) [20] and block neuromuscular transmission (100 µM for 35 min) [21]. Importantly, we recently found that acute chloroquine treatment diminishes maximal forces generated by the diaphragm muscle, likely by affecting neuromuscular transmission, which is generally consistent with aging effects in the diaphragm muscle [22]. In addition, we previously showed that aging impairs autophagy in phrenic motor neurons, reflected by increased expression of autophagy markers LC3 and p62 [16]. Accordingly, we hypothesized that chloroquine treatment increases the presynaptic retention of the styryl dye FM 4-64 following high-frequency repetitive nerve stimulation, consistent with the accumulation of unprocessed bulk endosomes.

## 2. Results

Images from 16 animals (25 ± 1 months of age) were used for analysis (eight females, eight males). The mean weight was 35 ± 6 g in females and 35 ± 3 g in males. There was no significant difference in weight across sexes (mean difference = −0.01, DF = 1365, t = −0.04, *p* = 0.97).

### 2.1. Morphological Measurements of Diaphragm Neuromuscular Junctions

Neuromuscular junctions sampled in each hemidiaphragm were en face and superficially located. Table 1 provides information on the number of animals and neuromuscular junctions sampled for each treatment, stimulation, or sex group, and the morphological measurements. A total of 1368 neuromuscular junctions were analyzed (*n* = 694 neuromuscular junctions from chloroquine-treated, 674 neuromuscular junctions from vehicle-treated, and on average 49 ± 18 neuromuscular junctions were sampled per hemidiaphragm).

The labeling of postsynaptic structures (labeled by α-bungarotoxin) was robust across all conditions, permitting morphological analyses at diaphragm neuromuscular junctions. There was no effect of treatment (F_1,1303_ = 3, *p* = 0.08) but a significant effect of stimulation (F_1,332_ = 15, *p* < 0.01) on the postsynaptic area of diaphragm neuromuscular junctions. No effect of sex (F_1,11_ = 2, *p* = 0.24), stimulation × sex interaction (F_1,332_ = 1, *p* = 0.43) or treatment × sex interaction (F_1,1303_ = 0, *p* = 0.60) was observed. There was a significant effect of treatment × stimulation interaction (F_1,1355_ = 12, *p* < 0.01). There was no effect of treatment × stim × sex (F_1,1355_ = 0, *p* = 0.62), but a significant effect of animal (*p* = 0.03) on the postsynaptic area of neuromuscular junctions was observed. The mean postsynaptic area in nonstimulated hemidiaphragms was 302 ± 60 µm^2^ in females and 298 ± 67 µm^2^ in males of the chloroquine-treated hemidiaphragm, and 332 ± 89 µm^2^ in females and 305 ± 72 µm^2^ in males of the vehicle-treated hemidiaphragm. In stimulated hemidiaphragms, the mean postsynaptic area was 292 ± 70 µm^2^ in females and 345 ± 95 µm^2^ in males of the chloroquine-treated hemidiaphragm, and 290 ± 92 µm^2^ in females and 326 ± 89 µm^2^ in males of the vehicle-treated hemidiaphragm (Table 1). Overall, the postsynaptic area of neuromuscular junctions sampled in the chloroquine-treated hemidiaphragms that were non-stimulated was ~3% smaller compared to the stimulated hemidiaphragms.

### 2.2. Frequency Dependent Increase in FM 4-64 Intensity

**Figure 1:**
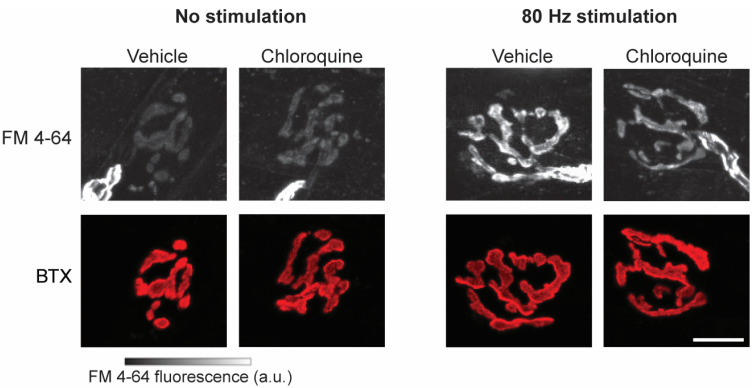
Representative mouse diaphragm muscle neuromuscular junctions across treatment and stimulation groups. Presynaptic structures were labeled by FM 4-64 (white, **top** panes) with uptake showing varying grayscale intensity (gradient scale bar on **bottom**). Postsynaptic structures were labeled by α-bungarotoxin (BTX; red, **bottom** panes). As expected, increased FM 4-64 fluorescence intensity is evident following high frequency (80 Hz) phrenic nerve stimulation in both vehicle and chloroquine-treated hemidiaphragms compared to the nonstimulation group. Note, labeled axons are also visible with FM 4-64 labeling since its high lipophilicity labels myelin sheaths at nerve endings. Scale bar, 10 µm.

Labeling of presynaptic structures (labeled by FM 4-64) at diaphragm neuromuscular junctions was evident in all conditions (Figure 1). There was no overall effect of treatment on FM 4-64 intensity at diaphragm neuromuscular junctions (F_1,1356_ = 0, *p* = 0.87). As expected, uptake was more pronounced following phrenic nerve stimulation, and there was minimal FM 4-64 uptake noted in the absence of stimulation. Accordingly, there was a significant effect of stimulation (F_1,1287_ = 1110, *p* < 0.01) on FM 4-64 intensity. No significant effects were observed for sex (F_1,14_ = 1, *p* = 0.28), treatment × sex interaction (F_1,1356_ = 0, *p* = 0.22) or stimulation × sex interaction (F_1,1287_ = 2, *p* = 0.15). There was a significant effect of treatment × stimulation interaction (F_1,1347_ = 148, *p* < 0.01), treatment × stimulation × sex interaction (F_1,1347_ = 21, *p* < 0.01) and animal (*p* = 0.01) on FM 4-64 intensity at diaphragm neuromuscular junctions. The overall mean FM 4-64 intensity in neuromuscular junctions was 552 ± 259 a.u. in nonstimulated hemidiaphragms and 1810 ± 635 a.u. in 80 Hz stimulated hemidiaphragms.

### 2.3. Chloroquine Increases FM 4-64 Uptake with No Stimulation

**Figure 2:**
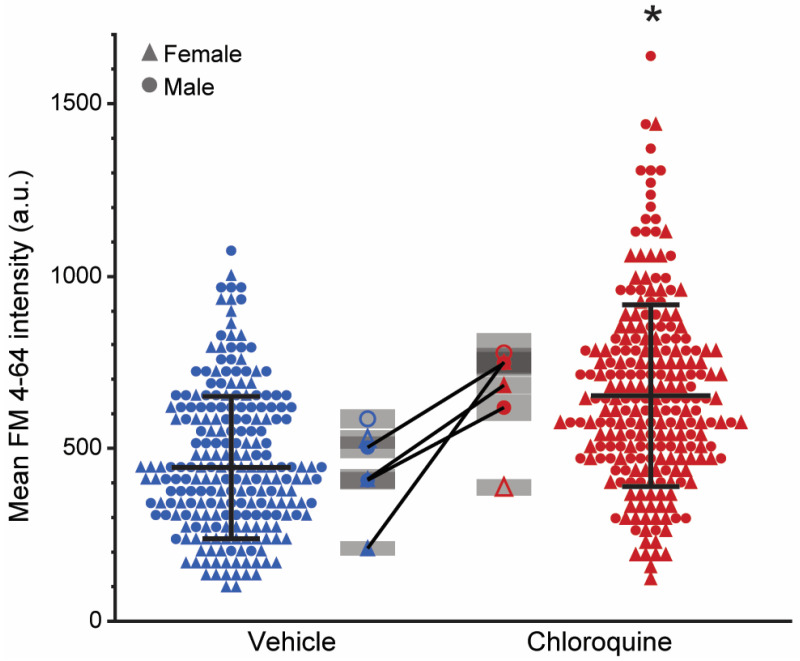
Mean FM 4-64 intensity at diaphragm neuromuscular junctions following vehicle or chloroquine treatment in the no-stimulation group. The scatter plots display FM 4-64 fluorescence intensity of individual neuromuscular junctions from vehicle (far left, blue) and chloroquine (far right, red) groups. Each point represents the FM 4-64 intensity of a single neuromuscular junction. The center line and error bars represent the mean and standard deviation for the entire group. In nonstimulated neuromuscular junctions, FM 4-64 was higher in the chloroquine group (F_1,452_ = 165, *p* < 0.01). Shaded plots positioned in the center next to each scatter plot represent the averaged values per hemidiaphragm with the mean FM 4-64 intensity for each hemidiaphragm as points and the standard error for neuromuscular junctions in that hemidiaphragm in the shaded bars. The solid black lines connecting points represent the hemidiaphragms from the same animal where one was treated with vehicle and the other with chloroquine. Symbols represent females and males as triangles and circles, respectively. *, *p* < 0.05 effect of treatment.

In the nonstimulated neuromuscular junctions, there was a significant effect of treatment (F_1,452_ = 165, *p* < 0.01, and no effect of sex (F_1,6_ = 2, *p* = 0.27) was observed. There was an effect of treatment × sex interaction (F_1,452_ = 11, *p* < 0.01) but no effect of animal (*p* = 0.10) (Figure 2). The FM 4-64 intensity was 653 ± 263 a.u. in the chloroquine-treated hemidiaphragms and 445 ± 207 a.u. in the vehicle. In four animals, one hemidiaphragm was incubated in chloroquine and the other side in the vehicle to assess the impact of chloroquine on FM 4-64 intensity within an animal (*n* = 154 chloroquine-treated neuromuscular junctions, 138 vehicle-treated neuromuscular junctions; average 37 ± 6 neuromuscular junctions per hemidiaphragm). In agreement with the overall effects, there was a significant difference in FM 4-64 intensity between the chloroquine and the vehicle-treated hemidiaphragm in all four animals (connecting lines in Figure 2), with a mean difference of 211 to 540 a.u. (DF = 61 to 81, t = −12.3 to −4.3, *p* < 0.01).

### 2.4. Chloroquine Decreases FM 4-64 Uptake at High-Frequency Stimulation

**Figure 3:**
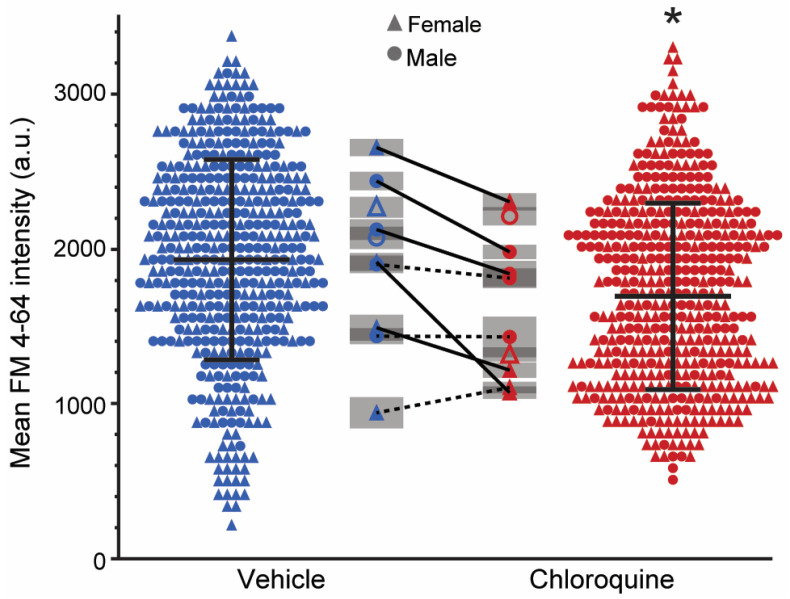
Mean FM 4-64 intensity at diaphragm neuromuscular junctions following vehicle or chloroquine treatment in the 80 Hz stimulation group. The scatter plots display FM 4-64 fluorescence intensity of individual neuromuscular junctions from vehicle (far left, blue) and chloroquine (far right, red) groups. Each point represents the FM 4-64 intensity of a single neuromuscular junction. The center line and error bars represent the mean and standard deviation for the entire group. After stimulation, high levels of FM 4-64 intensity were observed in both groups compared to no stimulation; however, the chloroquine-treated group displayed significantly lower FM 4-64 intensity compared to the vehicle group (F_1,903_ = 73, *p* < 0.01). Shaded plots positioned in the center next to each scatter plot represent the averaged values per hemidiaphragm with the mean FM 4-64 intensity for each hemidiaphragm as points and the standard error for neuromuscular junctions in that hemidiaphragm in the shaded bars. The black lines connecting points represent the hemidiaphragms from the same animal where one was treated with a vehicle and the other with chloroquine (solid and dashed lines represent statistically significant and non-significant differences, respectively). Symbols represent females and males as triangles and circles, respectively. *, *p* < 0.05 effect of treatment.

In diaphragm neuromuscular junctions following 80 Hz stimulation, there was a significant effect of treatment (F_1,903_ = 73, *p* < 0.01) with FM 4-64 intensity being lower in the chloroquine-treated hemidiaphragms compared to the vehicle (Figure 3). There was no effect of sex (F_1,10_ = 1, *p* = 0.27), or treatment × sex interaction (F_1,903_ = 2, *p* = 0.12). However, a significant animal effect was observed (*p* = 0.03). The FM 4-64 intensity with 80 Hz stimulation was 1694 ± 601 a.u. in chloroquine-treated hemidiaphragms and 1930 ± 646 a.u. in the vehicle group. A subset of eight animals had one hemidiaphragm incubated in chloroquine and the other side in the vehicle (*n* = 376 chloroquine-treated neuromuscular junctions, 365 vehicle-treated neuromuscular junctions; average 46 ± 12 neuromuscular junctions per hemidiaphragm) and are shown by the connecting lines in Figure 3. In five out of eight animals (solid lines), chloroquine treatment significantly decreased the average FM 4-64 intensity, with a range of reduction of 13% to 44% (mean difference = −273 to −842 a.u., DF = 73 to 121, t = 2.71 to 12.20, *p* < 0.01). In the remaining three animals (dashed lines), there were no significant differences in the average FM 4-64 intensity between treatment groups (mean difference = −89 to −163 a.u., DF = 69 to 94, t = −1.58 to 1.06, *p* > 0.12).

In order to evaluate synaptic vesicle density in the vicinity of release sites, mean FM 4-64 fluorescence intensity in the regions containing the greatest FM 4-64 fluorescence (reflecting 10% of the presynaptic terminal area) were analyzed in 297 neuromuscular junctions (155 chloroquine-treated neuromuscular junctions, 142 vehicle-treated neuromuscular junctions; average 19 ± 3 neuromuscular junctions per hemidiaphragm) randomly selected from animals that received stimulation on both hemidiaphragms (*n* = 8). In general agreement with the effects at the full presynaptic terminal, there was a significant effect of treatment (F_1,287_ = 18, *p* < 0.01), with chloroquine treatment reducing FM 4-64 intensity in these regions compared to the vehicle treatment. There was no effect of sex (F_1,6_ = 1, *p* = 0.32), treatment × sex interaction (F_1_,_287_ = 4, *p* > 0.05), or animal (*p* = 0.09). The mean FM 4-64 intensity in regions with the greatest FM 4-64 fluorescence intensity was 2931 ± 908 a.u. in the chloroquine-treated group and 3258 ± 948 a.u. in the vehicle-treated group. In four out of eight animals, FM 4-64 intensity in these regions was reduced on the chloroquine-treated side (mean difference = −201 to −1182 a.u., DF = 26 to 42, t = 2.70 to 6.33, *p* ≤ 0.01), with a mean intensity of 2877 ± 953 a.u. compared to 3588 ± 654 a.u. on the vehicle side. In the remaining four animals, there was no significant difference in FM 4-64 intensity in these regions (mean difference = −385 to 96 a.u., DF = 30 to 38, t = −0.90 to 1.87, *p* = 0.07 to 0.66), with a mean FM 4-64 intensity of 2982 ± 865 a.u. in the chloroquine-treated group and 2899 ± 1083 a.u. in the vehicle group.

## 3. Discussion

Chloroquine is commonly used in experiments to mimic autophagy inhibition, a mechanism increasingly recognized as important in aging disorders. In the present study, acute chloroquine treatment significantly decreased presynaptic retention of FM 4-64 at diaphragm neuromuscular junctions following 80 Hz phrenic nerve stimulation in old mice, consistent with disrupted synaptic vesicle recycling. In support, chloroquine treatment decreased FM 4-64 fluorescence intensity in regions with the greatest FM 4-64 fluorescence intensity comprising 10% of the total presynaptic area and which most likely surround synaptic vesicle release sites [10,23,24]. Disrupted synaptic vesicle recycling may interrupt bulk endocytosis indirectly. Retrieval of the presynaptic terminal membrane following high-frequency stimulation involves bulk endocytosis [25,26,27]. Accordingly, disrupted synaptic vesicle release at high-frequency stimulation with chloroquine treatment likely constrains bulk endocytosis and the internalization of bulk endosomes [26,28]. In the absence of stimulation, chloroquine treatment increased FM 4-64 fluorescence intensity in presynaptic terminals, consistent with disrupted processing of internalized membrane compartments. Taken together, these findings support an important role for autophagy in regulating synaptic vesicle recycling and processing of endosomal compartments at the presynaptic terminal and provide a basis for the role of autophagy impairments in age-related neuromuscular dysfunction.

Several recent studies support the role of impaired autophagy in diaphragm motor units. We recently reported that cervical motor neurons, including phrenic motor neurons, displayed increased expression of autophagy markers LC3 and p62 in old mice [16] and that acute chloroquine treatment in old mice (50 mg/kg for 4 h) diminishes the maximal transdiaphragmatic pressure generated during bilateral phrenic nerve stimulation, likely by affecting neuromuscular transmission [22]. Accordingly, the present study provides evidence for increased susceptibility to impairments in autophagy in older mice, resulting in disrupted neuromuscular transmission and, ultimately, force generation. These mechanisms are also likely at play for other motor systems, as we recently reported similar increases in LC3 and p62 markers in lumbar motor neurons [17], and are consistent with findings from motor neurons [29,30] and other neural systems in various neurodegenerative conditions [31,32].

In the present study, the styryl dye FM 4-64 was used to label the presynaptic terminal membrane and any internalized membrane following high-frequency stimulation. As expected, increased FM 4-64 fluorescence intensity was evident at diaphragm presynaptic terminals in both the vehicle and chloroquine treatment groups following 80 Hz stimulation. This suggests that synaptic vesicles and endosomes are labeled at this high-frequency stimulation, aligning with the expected induction of bulk endocytosis. However, in conditions without stimulation, FM 4-64 fluorescence specifically highlights synaptic vesicles, as bulk endosomes are typically not generated without high-frequency input. These findings underscore the effectiveness of FM 4-64 in visualizing synaptic activity and endocytosis processes. The differential labeling patterns observed under stimulated and nonstimulated conditions provide valuable insights into the dynamics of synaptic vesicle cycling and endocytosis in response to varying neural activity levels. In agreement, we previously showed increased cholera toxin subunit B (CTB) uptake in neuromuscular junctions following 80 Hz stimulation compared to 20 Hz stimulation [25]. The increase in lipid raft uptake (labeled by CTB) reflects the transition from endocytic mechanisms primarily reliant on clathrin-mediated vesicle retrieval to bulk endocytosis [26,27]. In the present study, disrupted endosomal trafficking following chloroquine treatment was evident in the absence of stimulation, where it is expected that only synaptic vesicles participating in spontaneous neurotransmitter release corresponding to miniature endplate potentials were labeled by an increase in FM 4-64 uptake [33]. The nature and extent of endosomal trafficking during nonstimulated conditions is poorly understood. Regardless, under high-frequency stimulation, bulk endocytosis pathways play an important role in the trafficking of synaptic membranes [25,26]. Chloroquine treatment reduced FM4-64 uptake during 80 Hz stimulation, suggesting disrupted presynaptic membrane retrieval. These findings suggest that the interplay between autophagy and synaptic vesicle or endosomal trafficking is of significant interest and should be the subject of further study.

Synaptic vesicle recycling is an important process that facilitates the reuse of synaptic vesicle membranes and proteins to maintain synaptic transmission, particularly during repetitive stimulation [34]. Four main mechanisms of synaptic vesicle recycling have been identified, including (1) clathrin-dependent vesicle recycling, which retrieves single vesicles to replenish the synaptic vesicle pool; (2) kiss-and-run, which reflects transient vesicular fusion and partial release of its contents; (3) “ultrafast” endocytosis, which reflects transient fusion and direct retrieval of vesicles; and (4) bulk endocytosis, which is defined by the retrieval of large endosomes directly from the presynaptic terminal membrane. Bulk endocytosis is predominantly observed during high-frequency stimulation. The internalized endosome requires further processing for recycling of synaptic vesicle components and replenishment of the synaptic vesicle pools [28].

Endosomal processing is characterized by molecular markers associated with different stages [35], and where Rab GTPases ensure the correct delivery of synaptic vesicle cargos to their intended destinations [36]. A rapid recycling pathway mediated by Rab4 allows membrane proteins and other endosomal contents to be assimilated back into the plasma membrane. Endosomal contents may also transfer to Rab11-containing recycling endosomes returning to the plasma membrane in a slower pathway or join early endosomes containing Rab5 and mature into late endosomes where Rab5 is replaced by Rab 7 [37]. These late endosomes can either mature into lysosomes, fuse with other late endosomes or existing lysosomes, and degrade their contents [38]. Lysosomes also receive cargo through fusion with autophagosomes during the process of autophagy activation [35]. There is emerging evidence of the role of autophagy in synaptic vesicle recycling [39,40,41]. Indeed, the sorting and degradation of synaptic proteins and the repair of damaged membranes subsequently require the endosomal sorting complex required for the transport (ESCRT) pathway [42], likely mediated by interactions with Rab35-containing endosomes. However, the crosstalk between autophagy and the ESCRT pathway is not well understood. Regardless, based on the results of the present study, autophagy inhibition (using chloroquine to inhibit autophagosome-lysosome fusion in old mice) was sufficient to disrupt synaptic vesicle recycling and reduce FM 4-64 uptake at diaphragm neuromuscular junctions, suggesting that appropriate sorting and processing of synaptic vesicle components and proteins requires functional autophagy pathways, particularly during high-frequency stimulation in old age.

A growing body of evidence documents the role of autophagy in maintaining neuromuscular junction function and plasticity [43]. Autophagy is a multistep degradation process to recycle altered proteins and other dysfunctional cellular components [15]. The formation of autophagosomes at synapses helps with the degradation of synaptic vesicle components, regulating fusion with lysosomes [44]. Impairments in autophagy can lead to the accumulation of misfolded proteins, which can disrupt endolysosomal trafficking, with possible roles in neurodegenerative diseases [45] and aging [16,17].

The complex molecular machinery responsible for membrane fusion during synaptic vesicle release and recycling is shared with the processing of endosomes and internal membrane compartments. The SNARE (soluble N-ethylmaleimide–sensitive factor attachment protein receptor) proteins are a large family of membrane proteins that mediate intracellular fusion steps [46]. Vesicle-associated membrane proteins (VAMPs) are a category of SNARE proteins that are present on the surface of synaptic vesicles and are essential for recycling, trafficking, and fusion in neurons [47]. Synaptophysin and some SNARE proteins (e.g., synaptobrevin-2 and VAMP4) are crucial in both exocytosis and endocytosis during synaptic vesicle recycling in neurons [48] and are retrieved via bulk endocytosis [49]. In support, rapamycin-mediated inhibition of autophagy decreased synaptic vesicle number in mouse striatum [50]. These findings are consistent with an important interplay between autophagy and synaptic vesicle recycling, as reported in the present study at diaphragm neuromuscular junctions of old mice.

In the present study, disrupted synaptic vesicle recycling following chloroquine treatment during high-frequency stimulation was evident by decreased presynaptic retention of FM 4-64 (i.e., decreased FM 4-64 fluorescence intensity) at diaphragm neuromuscular junctions (~10%), both overall and at the sites of greatest FM 4-64 fluorescence intensity representing 10% of the presynaptic terminal area and are most likely synaptic vesicle release sites [10]. In the levator auris longus muscle of mice, presynaptic terminals show a high degree of colocalization of exocytotic and endocytotic sites following stimulation at 100 Hz, consistent with the importance of coupled mechanisms for synaptic vesicle release and recycling via bulk endocytosis [33]. Notably, the colocalization of exocytotic and endocytotic sites was not evident following stimulation at 40 Hz. The observed decrease in FM 4-64 uptake can thus reflect alternative effects of chloroquine on other processes beyond inhibition of autophagy. In mouse phrenic nerve–hemidiaphragm preparations, chloroquine was also shown to reduce motor end plate potential and quantal content release by nerve stimulation (≥50 µM for 45 min) [20] and block neuromuscular transmission (100 µM for 35 min) [21]. Whether these represent the direct effects of chloroquine on release processes or the indirect effects of impaired trafficking is yet to be elucidated. In summary, inhibition of autophagy disrupts synaptic vesicle recycling and trafficking following high-frequency stimulation, requiring bulk endocytosis in old mice.

## 4. Methods

### 4.1. Animals

A total of 16 C57BL/6 × 129 J mice (eight females, eight males) at ~24 months of age (75% survival) were used for the experiment. Mice were maintained at the Mayo Clinic with a 12-h light–dark cycle with free access to food and water, caged in groups by age and sex. Mice were anesthetized with intraperitoneal ketamine (100 mg/kg) and xylazine (10 mg/kg) and euthanized by exsanguination. The diaphragm muscle with intact phrenic nerves was removed by making a midline incision in the thoracic cavity and then carefully dissecting the diaphragm from the ribcage to preserve nerve integrity.

### 4.2. Experimental Group Assignment

For each mouse, the diaphragm muscle was cut into two hemidiaphragms which were placed in Rees–Simpson’s solution (containing 135 Na^+^, 5 K^+^, 2 Ca^2+^, 1 Mg^2+^, 120 Cl^−^, and 25 HCO_3_^−^, all in mM, pH 7.4) with 95% O_2_-5% CO_2_ at room temperature (26 °C). Each hemidiaphragm (with the phrenic nerve attached) was mounted and stretched ~1.5 times resting length on a silicone-coated dish. To ensure unbiased allocation, a randomization process was employed for the assignment of 16 animals into four experimental groups. Each of the two hemidiaphragms from every animal was assigned to a treatment (chloroquine or vehicle in a 1:1 ratio) or stimulation group (80 Hz or no stimulation in a 3:2 ratio). Accordingly, animals were assigned to groups to facilitate within-animal comparisons and the following groupings were obtained: eight animals received stimulation on both hemidiaphragms, with one hemidiaphragm treated with chloroquine and the other with vehicle; four animals received no stimulation, with one hemidiaphragm treated with chloroquine and the other with vehicle; two animals were treated on both hemidiaphragms with chloroquine, with one side receiving stimulation and the other not; and two animals received no treatment, with one side stimulated and the other not.

### 4.3. Neuromuscular Junction Labeling

**Figure 4:**
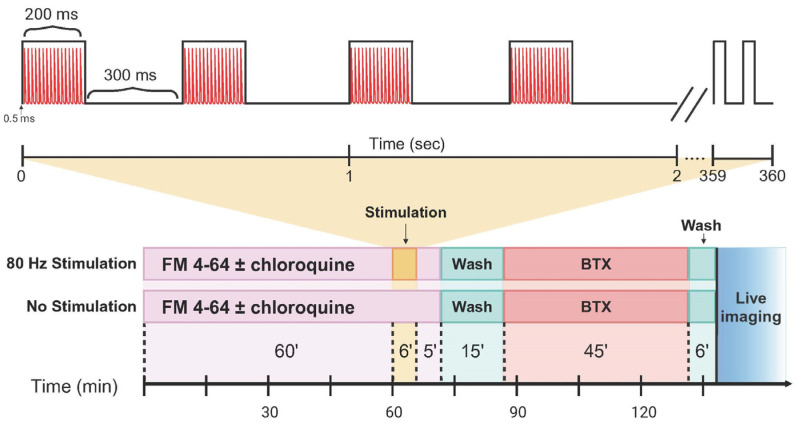
Experimental protocol for evaluation of FM 4-64 uptake at diaphragm neuromuscular junctions following repetitive nerve stimulation. Hemidiaphragm preparations were assigned to chloroquine (50 µM) or vehicle treatment, which was maintained for the duration of stimulation and poststimulation period. In the stimulation group (**top row**), hemidiaphragms and attached phrenic nerve were incubated in Rees–Simpson solution containing FM 4-64 for 60 min. The phrenic nerve was stimulated at 80 Hz in 200 ms trains of 0.5 ms supramaximal pulses delivered at 40% duty cycle for 6 min (inset above). A 5-minute poststimulation incubation while in FM 4-64 followed. In the nonstimulated group (**bottom row**), hemidiaphragms were incubated for 71 min to control for stimulation. In both groups, hemidiaphragms were then washed three times for a total of 15 min and then incubated in α-bungarotoxin (BTX) containing Rees–Simpson solution for 45 min followed by a 6 min wash before confocal imaging. Hemidiaphragms were oxygenated during the preparation at all steps through imaging.

Presynaptic structures at the neuromuscular junction were labeled by FM 4-64 (N-(3-triethylammoniumpropyl)-4-(6-(4-(diethylamino) phenyl)-hexatrienyl) pyridinium dibromide; Invitrogen, Waltham, MA, USA), a lipophilic, fluorescent styryl dye that integrates into the plasma membrane. During high-frequency stimulation, the dye is incorporated into synaptic vesicles and endosomes via endocytosis [33,51]. After stimulation, the tissue was extensively washed in Rees–Simpson solution to remove extracellular FM 4-64, leaving only the dye that had been internalized within the presynaptic terminal membranes. Each phrenic nerve-hemidiaphragm preparation was incubated in FM 4-64 (5 µM) in Rees–Simpson solution for one hour for presynaptic terminal labeling. For preparations assigned to the 80 Hz stimulation, the phrenic nerve was stimulated via a suction electrode (Grass S88 stimulator, Medical instruments, Guiney, MA, USA) using an Isolated Pulse Stimulator (model 2100, A-M Systems Inc., Carlsborg, WA, USA) in 200-ms trains of 0.5 ms supramaximal pulses delivered at 40% duty cycle for 6 min. Bulk endocytosis was induced by high-frequency stimulation while in the presence of FM 4-64, as previously reported [25,26]. All phrenic nerve-hemidiaphragm preparations were incubated in FM 4-64 and chloroquine/vehicle for an additional 5-minute poststimulation period [28] and subsequently washed three times (Figure 4).

Postsynaptic structures at the neuromuscular junction (i.e., motor endplates) were labeled using Alexa Fluor 488-conjugated α-bungarotoxin (peak excitation/emission: 495/519 nm; Molecular Probes, Eugene, OR, USA) by its binding to cholinergic receptors on the muscle membrane [8,9]. Hemidiaphragms were incubated in 1 µg/mL α-bungarotoxin in Rees–Simpson solution for an additional 45 min followed by a 6-minute wash. Neuromuscular junctions were imaged immediately following labeling while the preparation was kept in Rees–Simpson solution.

### 4.4. Confocal Imaging

All hemidiaphragms were imaged using an Olympus FluoView FV1200 laser scanning confocal system (Olympus America Inc., Melville, NY, USA) using a 40× (0.8 NA) water immersion lens. Motor endplates labeled by Alexa Fluor 488-α-bungarotoxin (peak excitation/emission 495/519 nm) and presynaptic terminals labeled by FM 4-64 (peak excitation/emission 515/640 nm) were imaged simultaneously using the 488 nm laser excitation, a 560 nm and 640 nm dichroic mirror, and 505–540 nm bandpass filters. Images were obtained at 12-bit resolution in a 1024 × 1024 array (0.2 µm × 0.2 µm) at 1.0 µm step size. All images were obtained the same day as the terminal experiment and within 4 h after stimulation. Only en face superficial neuromuscular junctions were selected for imaging and subsequent analyses [52]. Acquisition parameters (e.g., laser intensity, pixel dwell time, photomultiplier gain, voltage, offset, and confocal aperture) were consistent so that relative changes in fluorescence intensity could be reliably measured across experimental conditions.

### 4.5. Image Analysis

Confocal image stacks were used to obtain maximum projection images, which were subsequently processed and analyzed using a customized algorithm in MetaMorph (Molecular Devices, San Jose, CA, USA). Manual thresholding of the FM 4-64 (presynaptic) and α-bungarotoxin (postsynaptic) channels was performed separately to obtain the two binarized areas and determine the overlap. A region of interest was drawn manually around the borders of the presynaptic and postsynaptic overlap region to ensure the measurements were solely within the neuromuscular junction and unaffected by other FM 4-64 labeled components (e.g., axons). The overlap region was then used as a mask on the FM 4-64 channel to measure the mean FM 4-64 fluorescence intensity for each presynaptic terminal after background subtraction. In addition, mean FM 4-64 fluorescence intensity in the regions containing the greatest FM 4-64 fluorescence (reflecting 10% of the presynaptic terminal area) were also analyzed to evaluate synaptic vesicles in the vicinity of release sites, as previously reported [10,23,24].

### 4.6. Statistical Analysis

Data were analyzed using JMP 17.0.0 (SAS Institute Inc., Cary, NY, USA). The weight of animals was compared using a student’s *t*-test. Comparisons between groups for the postsynaptic area, background-subtracted mean FM 4-64 intensity (measured in arbitrary units, a.u.) of each neuromuscular junction, and the mean FM 4-64 intensity of the areas corresponding to 10% of the presynaptic terminal with the highest fluorescence intensity were analyzed using a mixed linear model with stimulation, treatment, and their interaction as fixed effects and animal as random effect. Based on previous data assessing FM 4-64 intensity at diaphragm neuromuscular junctions [10], we estimated that five animals per group would be sufficient to detect a 30% difference across treatment groups, with 80% power and α < 0.05. When appropriate, *post hoc* analyses were conducted using the Tukey–Kramer honestly significant difference test. Statistical significance was established at the 0.05 level. All experimental data are reported as mean ± standard deviation.

## Figures and Tables

**Table 1:** Diaphragm neuromuscular junction morphology. Mean postsynaptic areas of diaphragm neuromuscular junctions (labeled by α-bungarotoxin) across stimulation and treatment groups.

Stimulation	No Stimulation		80 Hz Stimulation	
Treatment	Vehicle	Chloroquine	Vehicle	Chloroquine
# Animals *	6	6	10	10
# of neuromuscular junctions	224	234	450	460
Postsynaptic area (µm^2^)	319 ± 83	300 ± 63	309 ± 92	318 ± 87

# Number, * Equal numbers of males and females in each group.

## Data Availability

The data supporting this manuscript are found within the text. Any additional data and the data that support the figures within this manuscript are available from the corresponding author upon reasonable request.
